# Bacteriospermia – A formidable player in male subfertility

**DOI:** 10.1515/biol-2022-0097

**Published:** 2022-08-17

**Authors:** Eva Tvrdá, Michal Ďuračka, Filip Benko, Norbert Lukáč

**Affiliations:** Department of Animal Physiology, Institute of Applied Biology, Faculty of Biotechnology and Food Sciences, Slovak University of Agriculture in Nitra, Tr. A. Hlinku 2, Nitra-Chrenová, 949 76, Slovakia

**Keywords:** bacteria, spermatozoa, oxidative stress, leukocytospermia, contamination, agglutination, immobilization

## Abstract

Bacterial colonization of male reproductive tissues, cells, and fluids, and the subsequent impact of bacteria on the sperm architecture, activity, and fertilizing potential, has recently gained increased attention from the medical and scientific community. Current evidence strongly emphasizes the fact that the presence of bacteria in semen may have dire consequences on the resulting male fertility. Nevertheless, the molecular basis underlying bacteriospermia-associated suboptimal semen quality is sophisticated, multifactorial, and still needs further understanding. Bacterial adhesion and subsequent sperm agglutination and immobilization represent the most direct pathway of sperm–bacterial interactions. Furthermore, the release of bacterial toxins and leukocytic infiltration, associated with a massive outburst of reactive oxygen species, have been repeatedly associated with sperm dysfunction in bacteria-infested semen. This review serves as a summary of the present knowledge on bacteriospermia-associated male subfertility. Furthermore, we strived to outline the currently available methods for assessing bacterial profiles in semen and to outline the most promising strategies for the prevention and/or management of bacteriospermia in practice.

## 1 Introduction

Scientific evidence gathered over the past decades strongly indicates that subfertility or infertility represents an increasing issue in the global scenario. This phenomenon is particularly notable in western countries, as revealed by epidemiological studies that show that male reproductive performance has declined by 1.5% per year in the United States [1,2]. Significant alterations to sperm quality have also been reported in European countries, such as Sweden [3], Denmark [4], Austria [5], Poland [6], France [7,8], and Italy [8,9]. Nevertheless, a progressive decrease in sperm quality over the past decades was also observed in Egyptian, Nigerian, Libyan, and Chinese males [10,11]. Despite a variety of currently available advanced diagnostic protocols, treatment, and management options for suboptimal male reproductive performance, infertility has become a significant issue in several species, including humans as well as farm animals [12].

Male infertility is defined as the male’s inability to achieve pregnancy in a fertile female following at least 12 months of regular, unprotected sexual intercourse [13]. As pointed out by Agarwal et al. [14,15], in humans, the cause of infertility lies exclusively with the male in 20–30% of all reported cases, while a male cause contributes to an additional 20% of infertile couples. As opposed to comprehensive reviews on the human species, large-scale reports are very sparse on farm animals. Nevertheless, existing studies indicate that subfertility is increasing in livestock as well [16], with male factor infertility accounting for 40–50% of an overall failure to successfully accomplish fertilization [17].

Fertility issues lead to increased social and psychological distress in humans [18] and present severe consequences on animal welfare and production [13]. Economically speaking, male infertility presents a significant financial burden on patients and the healthcare system [19]. The economic aspect of suboptimal male fertility management is even more amplified in farm animals since ejaculates from a single male may be used for artificial insemination in a large number of females, which is why semen samples from subfertile studs affect conception rates leading to considerable economic losses for farmers [13]. As such, a proper understanding of the causes of subfertility, early detection, and adequate intervention strategies may mitigate the negative consequences of suboptimal human or animal semen quality.

## 2 The role of bacteriospermia in the etiology of male infertility

Male infertility is a complex health issue that may be caused and/or aggravated by many causes or risk factors. The etiology of male infertility encompasses a wide range of hereditary or acquired causative agents that are often poorly understood, and their elucidation is often imprecise or highly subjective. According to Agarwal et al. [14], the origins of male subfertility may be classified into three general groups. Congenital or genetic causes encompass the cystic fibrosis gene mutation, numerical chromosomal abnormalities such as the Klinefelter syndrome, microdeletions of genes located on the Y chromosome, the Noonan syndrome, the Kallmann syndrome, or chromosomal translocations [20]. Acquired causes include a broad spectrum of factors ranging from traumatic injuries to the reproductive system, tumors, systemic diseases, varicocele to exogenous factors (heat, medication, surgical treatment, and so on), inflammation, oxidative stress, and sexual dysfunction [14,21]. Idiopathic risk factors more relevant to humans are represented by lifestyle choices, such as smoking, diet, stress, and exposure to toxins [22].

Among this large group of factors that may compromise the reproductive potential of males, bacteriospermia has emerged as a link between acquired and idiopathic aspects of male infertility and represents an important yet often-overlooked element that may compromise semen quality in humans as well as animals. Since the very first reports on agglutinating effects of *Escherichia coli* on spermatozoa [23] and isolation of *Brucella* from porcine semen published in the 1940s [24], more than 7,000 original papers have emerged to this date. Of these, over 2,700 research studies have been published over the past decade, indicating that the topic has been receiving increased attention from the medical, veterinary, and scientific communities.

Bacteriospermia is defined as the presence of bacteria in the seminal fluid [25] and is clinically acknowledged when bacteria in the ejaculate exceeds 1,000 colony-forming units (CFU)/mL [26]. The condition is often a consequence of acute or chronic bacterial infection of the male urogenital tract accounting for up to 15% of male infertility cases [26]. Various sites of the urogenital system may be affected by bacterial infection, including the prostate, epididymis, testes, and urethra [27], or the infection may be transmitted via sexual intercourse [28]. Bacteriospermia may be caused by both G^+^ and G^−^ bacteria and *Chlamydia* spp. or *Mycoplasma* spp. [25]. While the most common pathogenic bacteria identified as causative agents of urogenital infections and subsequent bacteriospermia are represented by *E. coli*, *Chlamydia trachomatis*, *Ureaplasma urealyticum*, *Mycoplasma*, *Staphylococcus aureus*, *Streptococci*, and *Enterococcus faecalis* [27], the male urinary system is not completely sterile as it has been already shown that certain bacteria, such as *Staphylococcus epidermidis*, are present in otherwise healthy subjects [29,30,31,32]. Furthermore, even in healthy individuals, semen may be contaminated by microorganisms during its passage through the genital tract, starting from the testes and expanding all the way to the prepuce and penile foreskin [33]. Particularly in the case of animals, bacteria present in ejaculates may originate from preputial fluids, skin, wool, urine, feces, or respiratory secretions [34]. While it is widely acknowledged that an individual with a good general health status produces semen of good quality [34], collecting and processing ejaculates are not antiseptic procedures. Hence, additional sources of semen contamination may include the artificial vagina, laboratory glassware, equipment, or semen extenders [32,33]. Moreover, contaminated feed and water, bedding, or poor hygiene standards may equally contribute to bacterial contamination of ejaculates [34,35].

**Figure 1:**
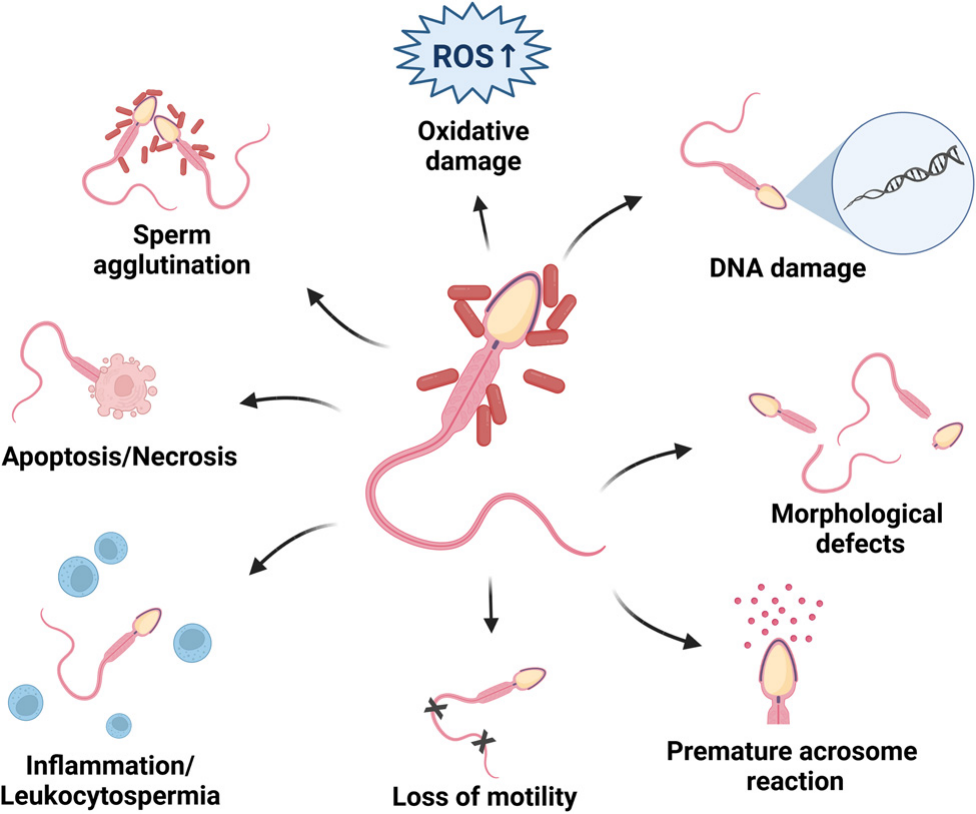
The effects of bacteriospermia on the sperm quality. The effects of bacteriospermia on sperm structure and function are distinct and multifactorial. Most reports have observed decreased sperm motility, alterations to the sperm morphology, and acrosomal degeneration. Frequently observed phenomena include DNA fragmentation and cell death. Sperm agglutination, oxidative stress, and a local immune reaction as a result of bacteriospermia have also been reported. Created with BioRender.com.

The effects of bacteriospermia on the resulting sperm quality are diverse and have been reported by a multitude of studies in several species including bulls [30,36,37], buffaloes [33], rams [31,35], boars [32,34,38], rabbits [39], turkeys [40], stallions [41,42], and humans [26,27,28,29,43]. Most of the studies agree that bacterial contamination of semen may lead to a decreased sperm motility and membrane stability [28,30,31,32,40,44]; alterations to the sperm head, mid-piece, and tail; acrosomal degeneration [28,30,31,32,45,46,47]; a stalled mitochondrial metabolism and ATP synthesis [30,31,40,47]; DNA damage; and phosphatidylserine dislocation [39,40,48,49]. Sperm agglutination [28,50,51], reactive oxygen species (ROS), overgeneration, and lipid peroxidation (LPO) [30,31,39,40] have been frequently associated with bacteriospermia as well. Bacteria present in ejaculates have been reported to trigger a local immune reaction often accompanied by leukocytospermia and secretion of cytokines [26,28,29,52], which have been often associated with a decline in male fertility. Finally, it has been suggested that bacterial infiltration into semen could modify the physicochemical or biochemical properties of the seminal plasma or semen extenders, which may compromise sperm survival *in vivo* as well as *in vitro* [32,45,53,54] (Figure 1).

Since bacterial infestation of semen is multivariable and complex, specific mechanisms of bacterial action on the quality of male reproductive fluids and cells will be discussed in detail.

### 2.1 Bacterial adherence

The adhesion process opens the doors for subsequent colonization of semen by bacteria. Bacterial adherence may be considered a crucial factor determining the invasive capability of bacteria and is proportional to the concentration of bacteria in the ejaculate.

An often-observed phenomenon is a bacterial adherence to the acrosome, leading to acrosomal disintegration that will arrest crucial fertilization mechanisms. As Zhang et al. [55] explained, bacterial adherence to the sperm surface may lead to an increased load of cells and thus impair sperm motion. Bacteria immobilized by adherence may furthermore attract other bacteria that will form complexes that agglutinate and form structures that may intervene with the motion of spermatozoa [56]. Subsequent agglutination may induce the secretion of extracellular polymeric substances and initiate biofilm formation [25,46,57]. Furthermore, sperm adhesion may trigger the release of exotoxins that might immobilize male gametes and alter their fertilization potential [58].

Bacterial adherence to host cells is a complex phenomenon that generally requires synchronized participation of different adhesion processes that may occur at the same time or at distinct stages of bacterial colonization. Bacteria able to adhere to the sperm surface contain polymeric adhesive fibers named “pili” or “fimbriae” that enabled first contact and subsequent infestation [59]. Pili are defined as virulence factors that present with the ability to mediate interbacterial aggregation and the formation of biofilms or to facilitate a specific recognition of host–cell receptors [58,59]. While it has been reported that pili play biological roles in the case of commensal microorganisms, the adherence affinity to spermatozoa has been well described in the case of *Enterococcus*, *Bacteroides*, *Bifidobacterium*, *Enterobacteriaceae*, and *Lactobacillus* [60].

The molecular mechanism of bacterial adherence is an intricate process that relies on the cooperation of pili, afimbrial adhesins, and interfacial free energy [55]. The phenomenon is furthermore facilitated by the fact that spermatozoa are, *per se,* rich in superficial glycoprotein receptors and are thus susceptible to bacteria–mediated interactions at the receptor-ligand level [61].

G^−^ bacteria, particularly *E. coli*, are known for their flagella and pili affinity to mannose receptors [56] that have been discovered on the sperm surface [62]. Type 1 fimbrinae, considered the most versatile virulence factor of uropathogenic *E. coli,* primarily mediate the attachment to the sperm surface and are involved in the promotion of the formation of intracellular bacterial communities and early stages of biofilm formation [56,61,63]. The essential receptor component in glycoproteins for type 1 fimbriae is a mannose group [25,56] which is located primarily in the sperm head. Another class of adhesins, specifically P fimbriae, is the widely studied mannose-resistant adhesion molecules observed in the majority of uropathogenic isolates [64], and *E. coli* strains are found in acute prostatitis [57]. The essential minimal active moiety in glycolipids for P-fimbriae is a-D-galp-l-4-9-D-galp (gal gal) [25] located predominantly in the sperm tail.

In the case of G^+^ bacteria, a common pilus is SpaCBA, which plays a role in bacterial colonization by binding to host cells, mucin, and mucous collagen and inducing bacterial aggregation. This pilus has been observed in *Lactobacillus* and *Corynebacterium*, exhibiting an exclusive sperm motility impairment without affecting the morphology or vitality of male reproductive cells [55,65].

Bacterial adhesion may be widely affected and promoted by afimbrial adhesins. According to Zhang et al. [55], a group of proteins called microbial surface components recognizing adhesive matrix molecules have been observed particularly in G^+^ bacteria. These molecules that are typical for *Staphylococci* covalently bind to peptidoglycans in the cell wall and target proteins in the host’s extracellular matrix [66]. Furthermore, they play essential roles in bacterial aggregation and induce a strong affinity to selected hydrophobic molecules [67]. Extra colonization benefits are provided by the so-called moonlight proteins that act as adhesins and have been observed in *Streptococcus*, *Lactobacillus*, and *Staphylococcus* isolates [55,68].

Bacterial adherence is significantly affected by inherent physical properties of the bacterial cell wall, such as hydrophobicity, charge distribution, and the area of contact, which are collectively defined as interfacial free energy. Bacterial adhesion is favored if free energy is negative, while positive free energy will act as a barrier between two cellular surfaces. Hence, the process of adherence will stall [69]. Appropriate free energy levels are crucial for the initial phase of bacterial adherence, during which bacteria attached to the sperm surface form a reversible and nonspecific adherence. A correct initial adherence then promotes a proper binding of the adhesins to the surface, leading to an irreversible time-dependent adhesion [70]. Matrix proteins, such as fibrinogen, fibronectin, and vitronectin, support the process of adherence since these act as additional adherent sites.

### 2.2 Sperm agglutination and immobilization

Sperm agglutination as a consequence of sperm–bacteria interactions may be defined as a process during which motile spermatozoa stick to each other—head-to-head, tail-to-tail, or mid-piece-to-tail. Nonspecific agglutination involving the adherence either of nonmotile spermatozoa to each other or of motile sperm to other cells, debris, or mucus threads may also occur [71].

This process is mediated by the inherent ability of bacteria to attach to each other. The high agglutination potential of bacteria leads to the creation of a more complex architecture called a biofilm, which ensures a more favorable environment for bacterial colonization. Intricate host–bacteria adhesions furthermore allow niches to be occupied by bacteria. At the same time, a solid layer of extracellular polymeric substances as a by-product of bacterial adherence decreases the entry of antibacterial molecules, complicating eventual treatment options for the infection [70,72]. Moreover, high bacterial density in the biofilm enables DNA cross-talks among bacteria, which may result in the spread of drug resistance patterns [72].

**Figure 2:**
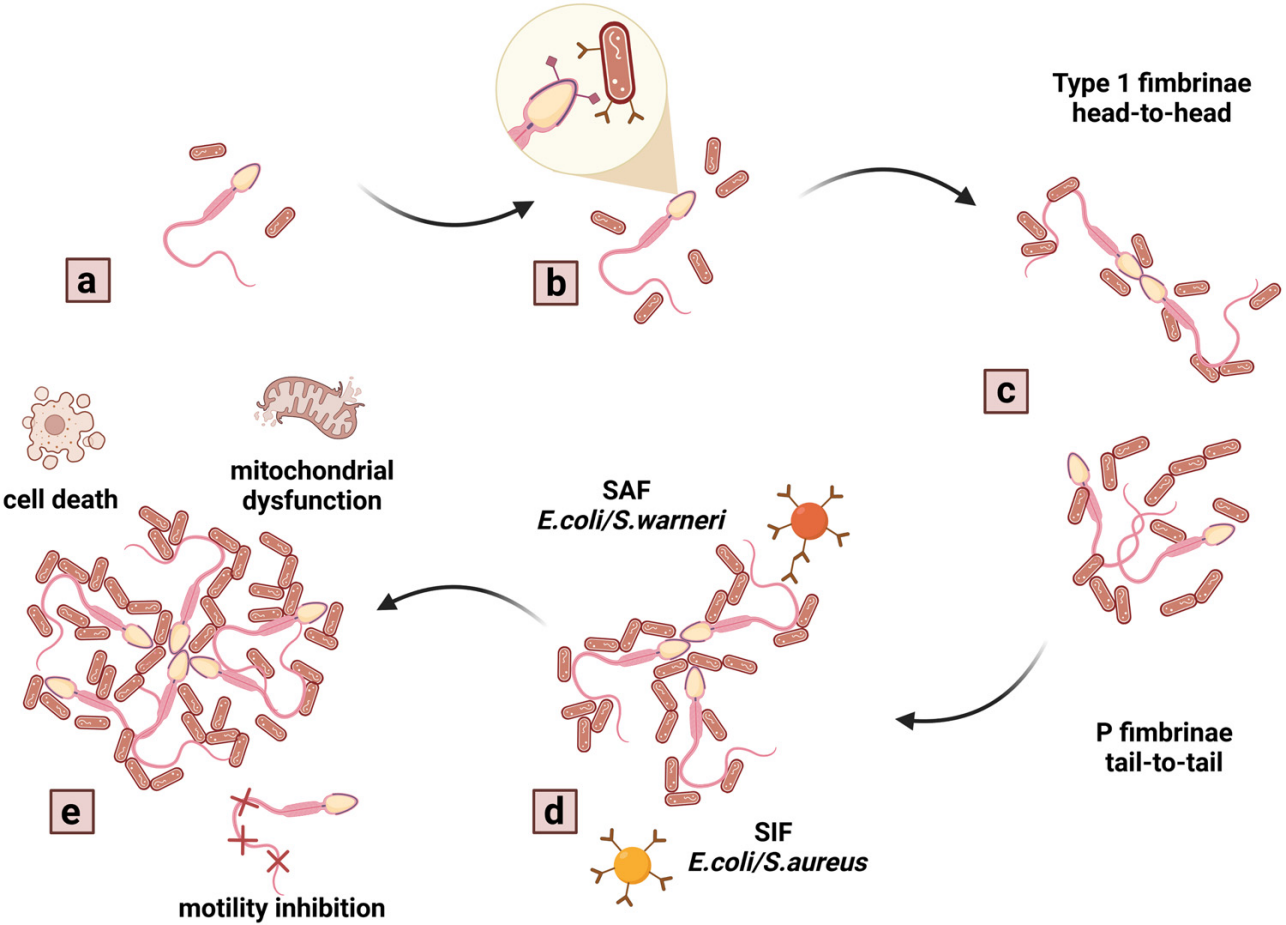
The process of bacteria promoted sperm agglutination. The agglutinating process as a result of bacterial presence in semen (a and b) relies on the type of pili that provides for the initial bacterial adherence to the corresponding sperm cell. Type 1 fimbrinae cause primarily head-to-head agglutination, while P-fimbriae are responsible for tail-to-tail agglutination (c). Subsequently, bacteria will release the SAF or the SIF (d) that will fortify the creation of biofilm (e), providing a more favorable environment for bacterial colonization. Inversely, spermatozoa affected by agglutination will exhibit signs of mitochondrial dysfunction, loss of motility, early onset apoptosis and/or necrosis (e). Created with BioRender.com.

The agglutinating process largely depends on the type of pili that catalyzes the initial bacterial adherence to the sperm cell. These fimbriae-dependent interplays may be competitively inhibited by the administration of a specific molecule found in the corresponding receptor. Type 1 fimbrinae which may be inhibited by mannose cause primarily head-to-head agglutination. In the meantime, P-fimbriae are inhibited by gal-gal and are responsible for tail-to-tail agglutination. A mixed agglutination is caused by bacterial strains that contain both types of pili, the activity of which may be inhibited by a mannose/gal-gal combination, supporting the theory that sperm agglutination is receptor dependent. Moreover, it has been suggested that spermatozoa contain a wide variety of superficial receptors, which is why even asymptomatic colonization of the male reproductive system by sperm agglutinating bacteria may lead to interactions that may evolve into agglutination of motile male gametes [59,61,66,69,71] (Figure 2).

As suggested by Monga and Roberts [61], host receptor variability and density on the sperm surface play a critical role in the host’s susceptibility to sperm agglutination. Proteins present in the seminal plasma and vaginal and cervical secretions may interfere with and/or mask receptor saccharides on the sperm surface, representing a barrier obstructing a potential interplay between the bacterium and sperm receptors for adhesion and/or agglutination, providing yet another important role of the reproductive fluids in the sperm protection during their transit within the urogenital system.

The most common molecule involved in the process of sperm agglutination is the sperm agglutinating factor (SAF), which has been isolated and purified from *E. coli* by Kaur et al. [73] and which blocks sperm motility by agglutination, causes morphological alterations in male gametes, and is spermicidal at higher concentrations. SAF interferes with the 125 kDa sperm receptor using its 71 kDa ligand that binds to both the sperm head and tail. Furthermore, a similarity of purified SAF to glutamate decarboxylase and receptor to major histocompatibility complex class I has been reported [74]. Receptor-specific interactions of SAF primarily target Mg^2+^-dependent ATPase that becomes competitively inhibited, as well as the surface receptors for cell death by apoptosis [73,74].

Subsequent studies on sperm agglutination also have isolated SAF-related molecules from other bacterial species. Pant et al. [75] uncovered an 80 kDa SAF molecule from *Staphylococcus warneri*, which also exhibited the ability to inhibit Mg^2+^ dependent ATPase activity and acted as a contraceptive in murine models. According to Ohri and Prabha [76], a 70 kDa protein is also produced by *S. aureus*, mediating a tail-to-tail agglutination of spermatozoa.

While sperm agglutination is a prime mechanism of sperm deterioration in the presence of *E. coli*, Paulson and Polakoski [77] revealed that this bacterium additionally secretes a small soluble ∼20 kDa protein that immobilizes spermatozoa without agglutinating them. Further tests have revealed that this sperm immobilization factor (SIF) is dialyzable and resistant to high or low temperatures. Follow-up studies have reported that *E. coli* also produces a thermolabile 56 kDa SIF, which recognizes a specific 113 kDa receptor located on the sperm membrane [51,78]. This molecule has detrimental effects on Mg^2+^-dependent ATPase activity and acrosome reaction induced by calcium ionophore [79].

A 20 kDa sperm immobilization protein isolated from *S. aureus* recognizes a specific 62 kDa receptor on the sperm surface, which – contrary to SIF from *E. coli – *could not be dialyzed nor withstand high temperatures [80]. Besides immobilizing spermatozoa in a fast and effective manner, SIF produced by *S. aureus* exhibited spermicidal effects on the sperm membrane, which was not observed in the case of *E. coli*-derived SIF [78,79,80].

The process of sperm agglutination and immobilization has been observed in the case of other uropathogens, such as *Mycoplasma*, *Chlamydia trachomatis*, and *Trichomonas vaginalis* [27,50,55,61,81]. Both processes are by and large simultaneous and reversible. However, high concentrations of other cytotoxic molecules released by bacteria may significantly impact the resulting vitality and fertilization potential of male reproductive cells. Spermatozoa affected by agglutination exhibit a high prevalence of morphological abnormalities and are prone to a premature acrosome reaction. Both phenomena have been markedly associated with the disruption of the activity of mitochondrial enzymes (particularly ATPase) crucial for sperm movement [26,27,47,79,82]. Consequently, the mitochondrial function is impaired, followed by an abrupt decrease of the mitochondrial membrane potential and subsequent rupture of mitochondria [28,47]. The resulting release of mitochondrial cytochrome C and ROS may lead to direct sperm apoptosis or necrosis, primarily responsible for decreased quality of semen [48].

### 2.3 Spermatotoxic bacterial products

Besides a direct contact of bacteria with spermatozoa, detrimental effects of bacteriospermia may be caused by extracellular molecules that are being synthesized and secreted by bacteria, such as lipopolysaccharide (LPS), hemolysins, or quorum sensing (QS) molecules.

LPS is a major component of the G^−^ bacterial cell wall [83]. During bacterial colonization, LPS is released and binds primarily to Toll-like receptor 4, stimulating pathogen-associated molecular pathways. Subsequently, nuclear factor-κB is activated to initiate the transcription of downstream inflammatory factors [84]. LPS has been frequently associated with reproductive toxicity [85,86], by interfering with the expression of pro-apoptotic genes [86,87]; however, recent studies have revealed that in contrast to the transcriptional machinery, LPS primarily affects the behavior of second messengers crucial for sperm function, such as the cyclic adenosine monophosphate (cAMP), Ca^2+^, and protein phosphorylation [88,89]. Li et al. [90] have unraveled that LPS reduced the intracellular cAMP of sperm independently of the levels of Ca^2+^ and protein–tyrosine phosphorylation. As such, the primary motility and penetration ability-inhibiting mechanism of LPS may be associated with the reduction of intracellular cAMP since this molecule is a crucial regulator of the sperm activity following ejaculation. Furthermore, as suggested by Zhang et al. [55], LPS-mediated regulation of sperm vitality may be accompanied by an increased generation of ROS and a subsequent disruption of the sperm membrane conformation.

Besides sperm agglutinating and immobilizing factors, uropathogenic *E. coli* encode a pore-forming toxin called α-hemolysin. α-hemolysin is a strong and ubiquitous cytolysin with the ability to form pores in the host cell membrane, which will ultimately result in cellular lysis [91]. The lysis process is independent of a receptor since α-hemolysin can permeabilize lipid bilayers of varying composition [92,93] and disrupt the colloidal osmotic pressure by forming voltage-dependent ion channels [93,94]. In the case of spermatozoa, hemolytic *E. coli* strains immobilize spermatozoa more efficiently and at a lower concentration in comparison with nonhemolytic counterparts. These can also induce a higher intracellular ROS production and a decline of the sperm mitochondrial membrane potential through cellular rupture [64,91,95]. Hence, α-hemolysin is released from the bacterial body and only exerts an effect if the bacterium adheres to the sperm surface [55].

Similar to α-hemolysin, β-hemolysin isolated from *Enterococcus* acts as a pore-forming membrane toxin that impacts the membrane integrity and thus contributes to sperm immobilization [55]. As observed by Qiang et al. [96], membranes of spermatozoa exposed to *Enterococci* and their toxic products were especially damaged on the head, neck, and the middle piece of the tail. Membranes covering the principal and the end piece of the tail were less damaged in comparison to the head. The head damage accompanied by the release of hydrolytic enzymes further confirms that the acrosomal region is the principal part of the sperm anatomy to be impacted by β-hemolysin and thus provides a link between enterococcal infection and male infertility.

The phenomenon of QS has gained substantial attention not only within the area of microbial communication in a predefined bacterial population but also in the field of interkingdom signaling and pathogenicity. QS is defined as the ability of microorganisms to “sense” their population density through a network of signaling molecules that are released and subsequently responded to. Once a QS concentration threshold is reached, these molecules will coordinate an array of activities, including biofilm formation, bioluminescence, and expression of virulence genes. Different QS molecules, such as the autoinducing peptides or *N*-acylhomoserine lactones, have been observed in numerous bacterial species such as *S. aureus* and *Pseudomonas aeruginosa* [46,97]. According to Rana et al. [33], soluble QS molecules of different bacterial origins may elicit diverse detrimental effects on male gametes. In this study, a reduction in sperm motility coincided in a dose-dependent manner with apoptosis and necrosis and a premature loss of the acrosome via a calcium-dependent mechanism. Since the male reproductive tract and cells are rich in communication receptors prone to interact with QS molecules, QS may become a new facet in the interaction of bacteria with male gametes and represents a putative link between bacterial communication systems and host infertility [46].

Finally, currently available evidence suggests an involvement of nonspecific enzymatic molecules produced by bacteria, such as coagulases, proteases, lipases, and coagulation factors that could play important roles in creating a favorable environment for successful bacterial colonization of male reproductive tissues and fluids [25].

### 2.4 Leukocytospermia as a cofactor of bacteriospermia

An inherent immune response to infection represents the infiltration of leukocytes to the source of inflammation. Leukocytospermia is acknowledged if the concentration of leukocytes positive for the peroxidase staining exceeds 1 × 10^6^/mL of semen, and the condition is generally linked to the presence of an infection and/or an inflammatory process in semen [26,29,52,98]. As suggested by Fraczek and Kurpisz [28], seminal white blood cells may be detected in the second phase of urogenital infection and are persistently present even following the elimination of the source of inflammation during the third stage of infection. This phenomenon is defined as isolated leukocytospermia [99,100]. However, other conditions may also lead to isolated leukocytospermia, such as varicocele, obesity, smoking, or traumatic injuries [97]. A definitive link between leukocytospermia and a decreased semen quality has to be reinforced yet, since some studies have emphasized a clear association between the presence of seminal leukocytes and alterations to the sperm concentration, motility, viability, DNA, and morphological integrity [26,29,30,31,32,40,101], while others revealed no effect of leukocytospermia on the fertilization potential, particularly with respect to artificial insemination or *in vitro* fertilization-associated conception rates [102,103].

**Figure 3:**
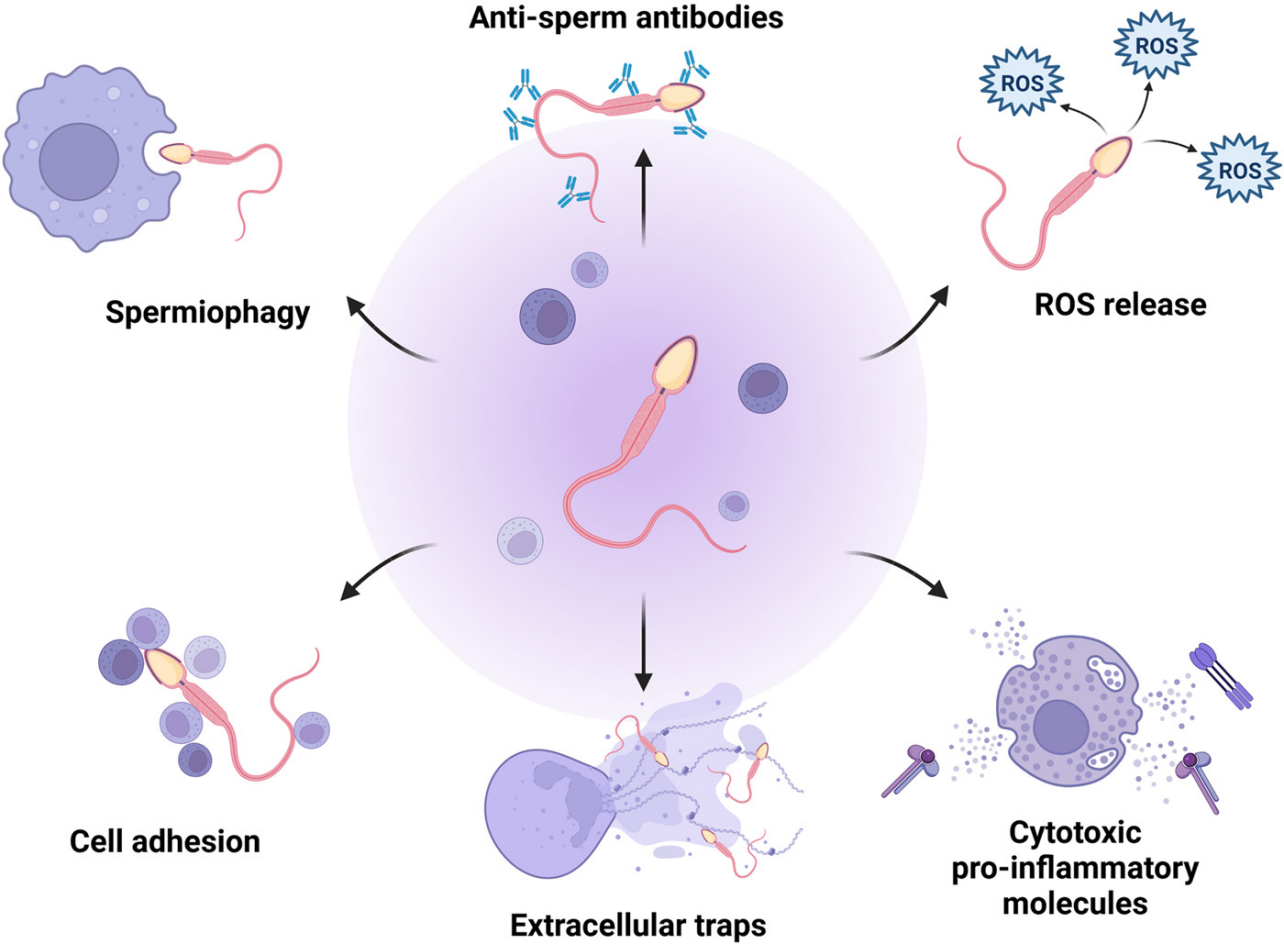
Leukocyte-inflicted damage to spermatozoa. The process may be directed through an array of processes: a direct attachment of the leukocyte to the sperm cell leading to the release of ROS and pro-inflammatory molecules, by phagocytosis (spermiophagy), by the creation of antisperm antibodies, and by ETs. Created with BioRender.com.

The Polish team of Fraczek and Kurpisz, regarded as pioneers in elucidating the molecular interplay of bacteriospermia and leukocytospermia, has postulated that leukocyte-inflicted damage to the male gamete may be directed through three processes: (a) a direct attachment to the cell, (b) by phagocytosis, and (c) by extracellular traps (ETs) [28] (Figure 3). Sperm deterioration through all proposed mechanisms of action may occur during leukocytospermia coexisting with bacteriospermia as well as during isolated leukocytospermia. Sperm damage inflicted by white blood cells is more severe during bacteriospermia, since within the innate defense mechanisms, leukocytes release an array of cytotoxic molecules, proteases, and ROS that may inflict further structural and/or functional damage to male reproductive cells [28,29,30,31,99,104].

The majority of white blood cells present in semen are represented by macrophages and polymorphonuclear neutrophils, which were originally thought to play a role in the surveillance and phagocytosis of abnormal and/or dead spermatozoa. Nevertheless, their inappropriate activation ignited by a tight adherence to male gametes leads to phagocytosis even of healthy spermatozoa [26,28,98,99,104]. In the first stage of spermiophagy, direct contact of both cells is followed by a tight adhesion of leukocytes to the surface of the sperm head, midpiece, or flagellum. This strong connection allows an entrapment, immobilization, and engulfment of spermatozoa by the cytoplasm of phagocytic cells. As observed by Piasecka et al. [105], active leukocytes formed cluster-like structures that most likely became the center of phagocytosis, capturing and engulfing spermatozoa, providing yet another example of leukocyte cooperation during bacteriospermia. The process is furthermore reinforced by the release of proinflammatory cytokines produced in large amounts by infiltrating macrophages, monocytes, lymphocytes, and dendritic cells [28].

Finally, all the aforementioned events may play a role in the disruption of immunotolerance and subsequent production of autoantibodies against sperm antigens. This pathophysiological phenomenon may be additionally fortified by molecular similarities between different bacterial strains and sperm antigens, as previously demonstrated by Prabha et al. [106] in the case of *S. aureus*, *E. coli*, *P. aeruginosa* , and *Proteus mirabilis* due to the existence of a common receptor for SIF on spermatozoa and bacteria. The outer core of bacteria often contains mannose, galactose, and *N*-acetyl glucosamine, which share analogies with carbohydrate moieties on the sperm surface [107], indicating a molecular resemblance between determinants present in male gametes and pathogenic bacteria. It seems that heat shock proteins (HSPs) also play a pivotal role in *Chlamydia*-associated molecular mimicry since these chaperones are released in response to stressful stimuli, such as in the case of bacterial infection [108,109]. The phenomenon is fortified by a 50% homology that is shared between the bacterial 60 kDa HSP family (bacterial HSP60), regarded as prime antigenic determinants during infection, and mammalian HSP60 [110]. The resulting cross-reactivity may lead to the development of inflammation and/or autoimmune reactions [111], which have been previously associated with male reproductive dysfunction through the induction of antisperm antibodies by seminal IgA antibodies or serum IgG antibodies [112]. However, the most recent studies are not unanimous regarding the association between the presence of chlamydial HSP antibodies and the presence of antisperm antibodies [108,112], with some authors reporting an association between them [108] in contrast to others [110,112].

A concomitant mechanism of an active immune response lies in the secretion of an array of cytokines, which present with the ability to inflict damage to male reproductive cells. As pointed out by Fraczek and Kurpisz [28], it is safe to assume that these biomolecules act within a network, which makes it difficult to define a specific spermatotoxicity of just one cytokine. As such, it seems plausible to hypothesize that the toxicity of one immunomolecule can be modulated in the presence of other molecular components of the immune system. Besides acting as prooxidants and inducing sperm damage primarily through LPO of the plasma membrane [104], it has been suggested that cytokines actively participate in the induction of the apoptotic machinery in ejaculated spermatozoa. Among different pro-inflammatory cytokines, tumor necrosis factor (TNF) α, one of the predominant cytokines released during inflammation and/or infection, is most often believed to act as an inducer of sperm apoptosis, phosphatidylserine translocation, or DNA fragmentation [113,114]. The proapoptotic behavior of TNF α may be further mediated via ROS or nitric oxide [114]. Within the large family of proinflammatory interleukins (ILs), IL-1b, IL-6, IL-8, IL-12, and IL-18 seem to play an important role in mediating inflammation-inflicted damage to male gametes. Their increased levels in response to bacterial overload in ejaculates have been correlated with a decreased sperm quality. It has been hypothesized that these immunomolecules could act as predictive biomarkers of ailments associated with bacteriospermia, such as prostatitis or male accessory gland infection (MAGI) [30,31,40,115,116,117]. Similar to TNF α, ILs are closely interconnected with an oxidative outburst [30,31,40] and a subsequent decrease in sperm motility is accompanied by an increased risk for DNA fragmentation [118,119].

The creation of ETs by activated leukocytes has been recently uncovered as a novel type of response by the immune system to the presence of infectious agents, which is catalyzed by the breakdown of the plasma membrane and a subsequent release of chromatin fibers following the disintegration of nuclear plasma (nuclear origin) or mitochondrial matrix (mitochondrial origin) [120,121]. While the backbone of ETs is composed of DNA and histones, the interior is embedded with a wide array of biomolecules such as lactoferrin, myeloperoxidase, defensins, bacterial permeability-increasing protein, proteases, or elastase, all of which present with significant antibacterial properties [121,122]. Contrary to the original beliefs that ETs were exclusive to polymorphonuclear neutrophils, macrophages, eosinophils, monocytes, and mast cells can also release ETs [123]. Depending on the cell type, ETs vary in shape and appearance, ranging from a diffuse appearance of fine chromatin fibers (such as in the case of monocytes) to a more spherical and compact decondensed chromatin (such as in the case of polymorphonuclear neutrophils) [121]. As unraveled by Schulz et al. [121] and Zambrano et al. [124], physical contact between a white blood cell and a sperm cell leads to rapid activation of the leukocyte, initiating ET formation. ET structures will then engulf the sperm head, midpiece, or flagellum, which is accompanied by the formation of small aggregates. ET fibers cause a physical blockage of male gametes, rapidly decreasing their motility. The process is further aggravated by the initiation of phagocytosis, degranulation, and activation of the cytokine communication network. Taken together, activation of the innate immune system as a response to infection or inflammation might lead to the formation of ETs, which will respond against their own male gametes as if these were recognized as potentially pathogenic agents. Since the release of ETs is a relatively newly discovered defense strategy, specific molecular mechanisms of action during the pathophysiological process of male subfertility need to be elucidated further.

### 2.5 Oxidative stress

Physiological and pathological roles of ROS in sperm physiology have become indisputable in affecting the male reproductive potential in health and disease. While low concentrations of ROS play an indispensable role in sperm maturation, capacitation, acrosome reaction, and fertilization, ROS overproduction and the resulting oxidative stress have been repeatedly observed in numerous male reproductive pathologies [14,21,22,28,58,104,114,125,126].

Bacterial contamination of semen has been frequently associated with increased oxidative pressure and an imbalance between ROS production and inherent antioxidant protective mechanisms of male reproductive cells, tissues, and fluids [125,126]. Generally speaking, the sources of ROS in bacteria-infested semen may be divided into three categories: (a) bacterial metabolism or products, (b) immune response and activated leukocytes, and (c) damaged spermatozoa.

The extent of ROS production by bacterial action by and large depends on a set of factors, such as the bacterial load and diversity, as well as the type of infecting, contaminating, or colonizing bacterial strain [28]. Aerobic metabolism of spermatozoa and aerobic and facultative anaerobic bacteria predestines them to produce ROS as their metabolic by-products [30]. Even anaerobic bacteria can deploy low-potential electron-transfer pathways, suggesting they be possible producers of reactive intermediates [127]. Superoxide, as well as hydrogen peroxide, have been reported to be released by a variety of potentially uropathogenic bacteria, such as *S. aureus* [128], *U. urealyticum* [129], *Bacteroides ureolyticus* [130], and *E. faecalis* [131], additional concentrations of which may contribute to the progression of oxidative damage to spermatozoa. As observed by Wang et al. [132], under *in vitro* conditions, several known pathogens and conditionally pathogenic species may be important inducers of oxidative stress responsible for the destruction particularly of the sperm membranes. Furthermore, virulence factors and toxic metabolites, such as LPS or hemolysins, may stimulate further ROS production by activated leukocytes.

The most predominant source of ROS are peroxidase-positive leukocytes, mainly polymorphonuclear neutrophilic granulocytes, which are activated during the inflammatory process [133]. Following infiltration of infectious agents, the initial immune reaction lies in an increase of seminal white blood cells [134,135]. A subsequent inherent response aimed to dispose of the pathogen results in an increased release of ROS from the activated leukocytes [135]. Two pathways have been suggested to play a pivotal role in the activation of seminal leukocytes during infection. The first lies in an increase of NADPH through the hexose monophosphate shunt. The second route is represented by a respiratory burst, which primarily acts as a protective mechanism during the infection [132,133,135]. Nevertheless, excessive infiltration and activation of seminal white blood cells can lead to concentrations of ROS that exceed a required level for normal physiological sperm functions, possibly leading to an impairment in the quality of semen, sperm concentration, and morphology [136].

According to Roca et al. [137], independently from naturally present abnormal sperm as a result of impaired spermatogenesis, large amounts of damaged or dead spermatozoa become a “silent killer” for viable male gametes. Deleterious effects of dead spermatozoa lie in the extracellular release of ROS (particularly hydrogen peroxide), which may cause irreversible damage to the plasma membrane of the viable sperm. Live spermatozoa affected by the release of the intracellular content from damaged or dead counterparts will then exhibit phosphatidylserine exteriorization and/or DNA breaks that would eventually lead to death by apoptosis or necrosis [137]. Necrotic cell death represents a more concerning phenomenon to the surrounding viable spermatozoa, as the process involves the loss of homeostasis by the rupture of the cell membrane and subsequent contamination of the extracellular environment by potentially spermatotoxic metabolites and the creation of sperm granulomas that may trigger an immune response [138,139].

Regardless of the sources of ROS, oxidative stress has become one of the leading causes of damage to the structural integrity and/or functional activity of the male reproductive cell. Sperm membranes are predominantly assembled of polyunsaturated fatty acids (PUFAs), which maintain a proper membrane fluidity, and are highly susceptible to oxidative overload [140]. Excessive amounts of ROS attack the double bonds present in PUFAs during the process of LPO, which will have a substantial impact on the semipermeable characteristics of the membrane, transport, and signaling processes, and enzymatic and receptor activities. Subsequently, the membrane loses its properties critical for normal sperm motion and fertilization [140,141]. The set of domino reactions involved in LPO will ultimately lead to the damage of DNA and proteins through the production of lipid peroxyl or alkoxyl radicals. Oxidative damage to the sperm DNA will occur primarily through base modifications, single and double-strand breaks, or crosslinks [142]. In the meantime, oxidative insults to proteins may result in amino acid modifications, the disintegration of the peptide chain, altered electric charge, and tolerance to proteolysis [143]. As reported by Mammoto et al. [144], increased levels of protein carbonyls in spermatozoa may lead to a decreased sperm capacity to penetrate the zona pellucida and obstructions in the sperm–egg binding and fusion process. Moreover, excessive ROS amounts may trigger the xanthine and xanthine oxidase system and downregulate the ATP synthesis, which may ultimately lead to a stall in sperm metabolism and lead to a subsequent cell death [125,140].

### 2.6 Cell death

Under physiological conditions, programmed cell death plays an essential role in assuring a selective deletion of male reproductive cells that have been affected by oxidative stress and are carrying fragmented DNA. Nevertheless, overactivation of the apoptotic cascade has been previously strongly associated with impaired sperm motion and morphology and with a decreased capability of spermatozoa to accomplish capacitation and successfully penetrate the ovum [145].

The potential involvement of bacteria in the promotion of apoptotic processes has been indicated by the number of studies revealing increased expression patterns of early and/or late apoptotic markers such as phosphatidylserine dislocation or TUNEL positivity in spermatozoa exposed to pathogenic as well as conditionally pathogenic bacterial species [39,47,49,146]. Furthermore, correlation studies on subjects with bacterial infection have observed an increased incidence of ultrastructural morphological changes typical of apoptosis or necrosis [39,40,147]. What is more, Fracek et al. [148] reported a simultaneous induction of complete apoptosis and necrosis in spermatozoa from normozoospermic subjects resulting only from simple contact with bacterial agents, even without the mediation of leukocytes.

Previous studies suggest that the principal pro-apoptotic mechanism of bacteriospermia may be linked to the interaction of bacterial endotoxins, such as porins, LPS, or peptidoglycans with Toll-like receptors 2 and 4, which are to be found on the sperm surface [149]. Exposure of spermatozoa to bacterial toxins may lead to an oxidative outburst and subsequent mitochondrial depolarization followed by rupture and caspase 3-mediated sperm apoptosis [150]. Furthermore, the immune response may be involved in sperm cell death through the cytokine network. Precisely, it has been speculated that IL-6, IL-8, IL-1b, or IL-18 could activate the Fas/Fas ligand complex located on the sperm membrane, followed by the initiation of caspase 8-driven cascade of events leading to DNA fragmentation and apoptosis [118].

### 2.7 Alterations to the biochemical milieu

Besides a direct spermatotoxic effect that bacteria and their products may exert on sperm biology, several studies that have recently emerged emphasize an indirect capability of bacteria to change the properties of the seminal plasma, thus rendering the medium to carry, protect, and nourish spermatozoa after ejaculation to be less favorable for their survival. As suggested by Ďuračka et al. [53] and Meena et al. [151], bacteria may compete for nutrients with spermatozoa as well as among each other, which is why a primary synergic effect of various opportunistic pathogenic bacteria may completely deprive nutrients present in the seminal plasma that otherwise would have been available for the sperm metabolism.

A correlation analysis revealed a significant negative association between the bacterial load and Mg levels in the seminal plasma. As Mg is essential for ATP synthesis, its decreased levels may be ultimately responsible for a decline in sperm motility [152]. Low Mg availability may further affect the activity of transaminases and phosphatases necessary for a proper sperm metabolism, which was confirmed by negative correlations among the activity of alanine transaminase, alkaline phosphatase, and bacterial load of semen, accompanied by a concomitant decrease of sperm motility [153]. The ability of bacteria to actively utilize nutrients at the expense of spermatozoa was also confirmed by an *in vitro* study, which revealed that a continuous decrease of magnesium and Ca available in a sperm culture medium by various *Staphylococcus* isolates was accompanied by the loss of sperm motility [49]. Accordingly, low levels of Ca and Mg have been previously detected in subjects suffering from reproductive ailments of different etiologies [154,155,156].

According to Ďuračka et al. [53], negative associations were also found between the presence of bacteria in semen and the levels of albumin or uric acid, which are major nitrogen compounds acting as secondary antioxidants and transport molecules ensuring a proper environment for an optimal sperm functionality [157].

Although preliminary, these pivotal findings strongly indicate that the bacterial load and/or diversity may modulate the seminal plasma composition, leading to insufficient or ineffective sperm nutrition and/or protection. As such, more studies are necessary to elucidate further side effects of bacterial metabolism on sperm survival.

## 3 Most important bacterial uropathogens

*E. coli* commonly inhabits the gastrointestinal tract of humans and warm-blooded animals, existing in commensal, symbiotic, and pathogenic relationships with its hosts. However, even commensal strains may promote a state of disease in immunocompromised organisms. A frequent occurrence of *E. coli* in semen makes it the most researched bacterium regarding suboptimal sperm quality. It appears that pili and flagella represent a key virulence factor of *E. coli*-associated pathogenicity toward spermatozoa. It has been previously shown that almost 75% of *E. coli* semen isolates matched urinary serotypes, while flagellar isolates prevailed [158]. Head-to-head sperm agglutination was observed in type-1 fimbriated strains because of mannose residues in the sperm head, while tail-to-tail sperm agglutination was noticed in P-fimbriated strains [61]. Nevertheless, different strains may affect different sperm structures or functions at different concentrations. Particularly, hemolytic *E. coli* strains pose a higher threat to sperm quality compared to non-hemolytic strains, even at lower concentrations [95].

*Staphylococcus* sp. has been identified in ejaculates stemming from humans [159], bulls [30], rams [31], boars [32], turkeys [40], roosters [160], stallions [42], and bucks [161]. Despite the fact that staphylococci belong to the most identified bacteria in semen, numerous species have been reported to act as normal components of the skin microflora. *S. epidermidis* has been previously isolated from 64% of human semen samples, while no changes in sperm motility were found [162]. Conversely, some studies emphasize the unfavorable effects of skin staphylococci on spermatozoa triggered by ROS overgeneration [30,163]. Currently, *S. aureus* belongs to the most researched species possessing several known factors of virulence, including enterotoxins, lipoteichoic acid, and toxic shock syndrome toxin 1. A further report indicates that *S. aureus* has the potential to avoid ETs by releasing nucleases and adenosine synthases. These enzymes convert ET structures to deoxyadenosine, which will induce apoptosis of immune cells through the activation of caspase 3 [164].

Even though *Enterococcus* spp. is listed among the characteristic representatives of gut microflora, an array of studies has provided evidence to suggest that this bacterium could act as an undesirable cause of male subfertility or infertility. A recent meta-analysis [165] summarized publications reporting on the presence of *Enterococcus* spp. in human semen, according to which 17 of 18 studies detected the presence of this bacterium in specimens from men attending fertility clinics. In addition, Moretti et al. [166] have revealed a significant decrease in sperm concentration and motility in patients who tested positive for *E. faecalis*. Similar to *E. coli*, flagella and pili play a major role in the pathogenicity of *Enterococcus* spp. to other cells and contribute to cell aggregation. In their study, Mehta et al. [167] speculated if direct cell-to-cell contact or metabolic products could be responsible for compromised semen quality in specimens carrying *E. faecalis*. Likewise, Villegas et al. [168] indicated that toxic metabolic products of *E. faecalis* released by their proliferative activity and direct contact increased the resulting cytotoxic effect. According to Fraczek et al. [148], *E. faecalis* was identified in 13.8% cases of healthy young normozoospermic donors with asymptomatic bacteriospermia. At the same time, the sperm quality parameters such as sperm concentration, membrane integrity, mitochondrial membrane potential, and DNA integrity were impaired when compared to the control group without any bacterial presence.

Previous studies have revealed that both healthy donors and patients with prostatitis faced the presence of coryneform bacteria in semen. Although Türk et al. [169] identified coryneform bacteria in 76% of patients suffering from prostatitis, the bacteria were also present in 83% of cases of the control group, which makes their presence in semen unpredictable toward the fertility potential. Generally, coryneform bacteria are considered to be commensals of the urethra or lower urogenital tract. An invasion of the upper urogenital tract and resulting prostatitis may turn their behavior into a saprophytic one. *Corynebacterium glucuronolyticum* (formerly known as *Corynebacterium seminale*) was previously reported to act as an opportunistic pathogen with an increased potential to cause male urethritis syndrome [170], monomicrobial paucisymptomatic bacterial prostatitis [171], or encrusted cystitis even without the presence of predisposing factors [172]. A few studies [65,173] have evaluated the impact of *C. glucuronolyticum* on basic semen parameters and stated that this bacterium was the most abundant species among the semen samples from infertile men. Meštrović et al. [170] designed a prospective pretreatment and posttreatment investigation with a strict criterion regarding the involvement of the semen specimens with a monoculture of *C. glucuronolyticum*. Their analysis showed a significantly increased percentage of spermatozoa with neck and mid-piece defects. Following treatment, only an improvement in sperm vitality was observed. Therefore, the effect of coryneform bacteria on sperm quality remains indefinite. Even though an array of studies has tried to express the involvement of specific bacterial species in the resulting sperm functionality, the majority of semen samples are still inhabited by a variety of different microorganisms, acting in a relatively synchronized and symbiotic manner.

*Lactobacillus*, the largest genus among bacteria presenting with the ability to produce lactic acid, has been defined as a nonpathogenic component of intestinal and urogenital floras. Generally, the presence of lactobacilli has not been associated with diseases. The principal role of *Lactobacillus* species in the vaginal tract is indisputably to maintain a physiologically normal vaginal microbiota and thus prevent possible colonization of foreign bacteria [174]. However, only a few studies have focused their attention on the effect of lactobacilli on sperm quality. Lactobacilli is able to adhere to mucous membranes. Nevertheless, the controversial question remains if these bacteria may adhere to the sperm surface and thereby impair the fertilization potential, as recently discussed by Zhang et al. [55]. Like other species, the concentration of lactobacilli is a key factor in their pathogenic or probiotic effects on spermatozoa. In other words, the higher the lactobacilli concentration, the greater their adherence to male gametes. Vaginal lactobacilli may act as a selector of the most viable spermatozoa during natural mating. Slowly moving spermatozoa or sperm cells with morphological aberrations will be “caught” and agglutinated. However, any bacterial intervention during artificial insemination may block successful fertilization. Particularly, the sperm DNA is susceptible to bacterial presence following thawing [175].

Baud et al. [43] studied the microbiota of human ejaculates, encompassing 26 normozoospermic and 68 samples with at least one abnormality. The authors observed that *Lactobacillus* prevailed in samples with normal sperm morphology. Similar results were observed by Weng et al. [176], who defined *Lactobacillus* as the most prevalent group, particularly in the samples accomplishing the criteria for normal semen quality. Interesting research was conducted by Barbonetti et al. [177], who analyzed whether a combination of three selected strains of *Lactobacillus* could prevent *in vitro* Fe^2+^-induced LPO. The authors selected *L. brevis*, *L. salivarius*, and *L. plantarum* strains, considering them as the prevailing bacterial representation on the surface of the vaginal mucosa, preventing urinary tract infections and antioxidant properties of lactobacilli. This study showed that the presence of *Lactobacillus* species at a concentration of 1 × 10^8^ CFU had the potential to restrain LPO and significantly maintain sperm motility and viability under induced oxidative stress.

Table 1 provides the strongest consensus effects of different types of bacteria across original reports published on the topic.

**Table 1:** Leading consensual effects of different bacterial species on the sperm structural integrity and functional activity

Effect	Bacterium/Bacteria	References
Inhibition of sperm motility	*C. trachomatis*	[81,178,179,180,181,182,183,184,185,195]
	*Mycoplasma* spp.	[178,184,186,187,188,189]
	*E. coli*	[50,146,166,168,188,189,190,191,192,193,194]
	*S. aureus*	[30,49,66,168,188,189,194,195,196,197,198,199,200,201,202,203]
	*U. urealyticum*	[179,188,189,195,204,205,206,207]
	*E. faecalis*	[39,166,201,208,209]
	*P. aeruginosa*	[207,210,211,212]
Damage to the sperm membrane	*E. coli*	[47,56,146,166,190,192,213,214,215,216,217]
	*Staphylococcus* spp.	[39,47,163,168,218]
	*Enterococcus* spp.	[96]
	*P. aeruginosa*	[207,210,211,212]
	*U. urealyticum*	[206,219,220]
	*C. trachomatis*	[81,150,182,221,222]
	*P. aeruginosa*	[207,210,212]
Mitochondrial dysfunction	*E. coli*	[47,50,146,192]
	*Staphylococcus* spp.	[30,47,49,197,163]
	*P. aeruginosa*	[207,212]
	*U. urealyticum*	[223,224]
Sperm morphology abnormalities	*E. coli*	[78,190,194,213,225,226]
	*Staphylococcus* spp.	[194,196,203,227]
	*E. faecalis*	[167,227]
	*U. urealyticum*	[179,228,229,230,231]
	*C. trachomatis*	[185,186,232]
	*Mycoplasma* spp.	[179,187,230]
DNA fragmentation	*Mycoplasma* spp.	[178,179,187,233]
	*E. coli*	[50,216]
	*Staphylococcus* spp.	[30,49,189]
	*C. trachomatis*	[150,178,233,234,235,236]
	*U. urealyticum*	[236,237,238]
Sperm agglutination/aggregation/immobilization	*E. coli*	[6,23,61,74,239,240]
	*Staphylococcus* spp.	[66,75,76,80,195,202]

## 4 Bacteriospermia in practice

Approximately 6.9–8% of sexually active men have been estimated to suffer from a urogenital infection [241]. Among the most commonly diagnosed ailments of the urogenital tract, chronic urethritis, prostatitis syndrome, epididymitis, and orchitis play a prime role. Furthermore, viral infections may contribute to chronic inflammation and thus decrease the fertility potential [242].

The World Health Organization (WHO) has published criteria to diagnose MAGI based on physical, urine, and semen examinations. In particular, medical history of any previous urinary tract infection, sexually transmitted infection, or epididymitis should demand attention. The physical examination findings include a thickened and tender epididymis, a thickened spermatic cord, and an abnormal digital rectal examination. Urine is collected following prostate massaging, while any abnormalities and the presence of *Chlamydia trachomatis* are being monitored. Positive findings from the semen evaluation include leukocytospermia, a positive semen culture for any pathogens, a positive *C. trachomatis* test, increased inflammatory markers or ROS levels, and any abnormalities in the biochemical profile of the seminal plasma. MAGI is diagnosed when two of three of the aforementioned findings are positive alongside oligozoospermia, and/or asthenozoospermia, and/or teratozoospermia [243]. Chronic prostatitis is characterized by at least 10 times higher bacterial count following a prostate massage. A significantly elevated bacterial count may occur in 50% of ejaculates from prostatitis patients, while leukocytospermia and increased concentrations of IL 8 are frequently observed as well [244].

Asymptomatic genital tract inflammation used to be mistaken for chronic prostatitis. This ailment differed from prostatitis by no changes in the volume and pH of semen, frequent damage to the flagellar membrane, no changes to the seminal zinc concentration, and decreased levels of α-glucosidase [244].

According to Schiefer [245] and Cottell et al. [246], common urethral commensals present in the ejaculate do not necessarily mean a genital infection. Therefore, a significant bacteriospermia was defined as 10^3^ CFU in 1 milliliter of ejaculate [247]. Leukocytospermia has been previously observed in bacteriospermic patients. Furthermore, Domes et al. [29] have associated the presence of neutrophilic granulocytes with suboptimal semen quality parameters, including sperm DNA integrity.

Semen examination, according to the WHO recommendations, is a crucial factor in detecting any infection or inflammation in the male urogenital tract. However, basic semen parameters do not have the strength to indicate infectious or inflammatory processes. Although volume, pH, and the biochemical profile of the seminal plasma are included in the differential diagnosis, they only provide information about the accessory glands and their function. The presence of IgG and IgA immunoglobulins in semen may indicate sperm agglutination. An ongoing inflammatory process is furthermore very well indicated by the presence of granulocyte elastase and proinflammatory cytokines [244].

Usually, urine samples are tested for the presence of urinary pathogens. Clinically, the presence of commensals in urine, e.g., *S. epidermidis* or *S. viridans*, is insignificant. Conversely, *Escherichia* sp*., Enterococcus* sp., *Proteus* sp., *Mycoplasma* sp., *Ureaplasma* sp*., Klebsiella* sp., and *Staphylococcus* spp. may cause an infection similar to obligatory pathogens [245]. Moreover, the result of the microbial profile may be affected by the transport, sampling, and processing time and by insufficient prepuce cleaning. It is also recommended to urinate before masturbation. According to recent recommendations, the most reliable results are obtained when both bacterial culture and Polymerase chain reaction (PCR)-based microbial analysis are performed. Sexually transmitted pathogens are difficult to cultivate on agar plates [244]. Table 2 displays currently published studies concerning bacteriospermic specimens, identified bacteria, and their effect on sperm quality parameters.

**Table 2:** Current studies concerning bacteriospermia, identified bacteria and their consequences on semen quality

Number of samples	Identified bacteria and their frequency	Effect on spermatozoa quality	References
*n* = 39 (infertile)	*S. haemolyticus* (38%)	↓Sperm concentration and progressive motility in case of *E. coli*, *U. urealyticum*, and *S. aureus*	[188]
	*Peptostreptococcus* (21%)		
	*E. faecalis* (20%)		
	*E. coli* (20%)		
	*Ureaplasma urealyticum* (17%)	↓Vitality in case of *U. urealyticum*	
	*Mycoplasma hominis* (9%)	↓Morphology in case of *S. aureus*	
	*S. aureus* (9%)		
	*Bacteroides fragilis* (2%)		
*n* = 454 (infertile, symptomatic chronic prostatitis)	*Chlamydia trachomatis*	↑ pH, ↓ sperm concentration, motility, morphology	[248]
*n* = 707 (fertile, symptomatic chronic prostatitis)	*Enterococcus* spp. (37%)	↑ pH	
	*S. saprophyticus* (26%)		
	*E. coli* (18%)		
	*Group B streptococcus* (10%)		
	*Klebsiella* sp. (7%)		
	*Serratia* sp. (2%)		
*n* = 118 (fertile, asymptomatic)	*Mycoplasma* sp. (22%)	↓Sperm concentration, total sperm count,	[249]
	*Ureaplasma* sp. (35%)	↓Motility and morphology in case of Mycoplasma	
	*Chlamydia* sp. (32%)		
*n* = 1,650 (infertile, asymptomatic)	*C. trachomatis* (1.6%)	↑Sperm DNA fragmentation in case of *U. urealyticum* and *M. genitalium*	[250]
	*M. genitalium* (3.1%)		
	*N. gonorrhoeae* (0.4%)		
	*U. urealyticum* (86%)		
	Mixed infection (8.9%)		
*n* = 60 (infertile, asymptomatic)	*E. faecalis* (25%)	↓Sperm concentration, total and progressive motility, viability, morphology	[251]
	*S. agalactiae* (16.7%)		
	*E. coli* (16.7%)	↑Sperm DNA fragmentation	
	*S. haemolyticus* (11.7%)	Leukocytospermia	
	*S. aureus* (8.3%)		
	*Proteus* spp. (6.7%)		
	*K. pneumoniae* (5%)		
	Multibacterial (10%)		
*n* = 29 (infertile, symptoms not specified)	*S. aureus* (27.6%)	Leukocytospermia	[200]
	*S. epidermidis* (17.2%)	↓Sperm concentration, total and progressive motility, fertilization rate	
	*S. haemolyticus* (13.8%)		
	*E. coli* (20.7)	Sperm protamine deficiency	
	*E. faecalis* (13.8%)		
	*S. agalactiae* (6.9%)		
*n* = 36 (infertile, asymptomatic)	*S. aureus* (38.9%)	↓Sperm motility, morphology	[252]
	*S. saprophyticus* (22.2%)		
	*E. coli* (16.7%)		
	*P. mirabilis* (8.3%)		
	*P. vulgaris* and *K. pneumoniae* (5.6% for each)		
	*P. aeruginosa* (2.8%)		
*n* = 1,200 (nonazoospermic subfertile, majority were asymptomatic)	*E. faecalis* (56%)	↑Sperm DNA fragmentation	[29]
	*E. coli* (16%)		
	Group B *streptococcus* (13%)		
	*S. aureus* (5%)		
	*K. pneumoniae* (2.2%)		
	*P. mirabilis* (1.7%)		
	*Citrobacter koseri* (1.5%)		
	*Morganella morganii* (1.3%)		
*n* = 28 (infertile, asymptomatic)	*E. faecalis* (30%)		[201]
	Coagulase-negative *Staphylococcus* (23.3%)		
	*S. aureus* (20%)		
	*E. coli* (10%)		
	*K. pneumoniae* and *Proteus* sp. (6.66% for each)		
	*Citrobacter* sp. (3.3%)		
*n* = 52 (fertile and normozoospermic, asymptomatic)	Coagulase-negative *Staphylococcus* (22.9%)	↓Sperm concentration, motility, morphology, membrane integrity	[42]
	*Streptococcus* (18.3%)	↓Viability and total sperm count only in case of Leukocytospermia	
	*E. faecalis*, *E. faecium* (13.8%)		
	*C. glucuronolyticum*, *C. striatum, C. propinquum* (16.5%)	↑Sperm DNA fragmentation	
	*E. coli, P. mirabilis* (3.7%)		
	Anaerobic G^+^ (6.4%)		
	Anaerobic G^−^ (13.8%)		
*n* = 31 (infertile, asymptomatic)	*C. trachomatis*	↓Sperm motility, concentration, morphology	[253]
	*M. hominis*		
	*U. urealyticum*		
*n* = 92 (infertile, asymptomatic)	*S. aureus* (28.3%)	↓Total sperm count, motility, morphology	[194]
	*E. coli* (19.6%)	Immobilization in case of *E. coli*	
	*S. saprophyticus* (13.0%)		
	*P. mirabilis, P. vulgaris*, and *Klebsiella* spp. (10.8% for each)		
	*P. aeruginosa* (6.5%)		
*n* = 60 (infertile, asymptomatic with leukocytospermia)	*C. trachomatis* (41.7%)	↑ pH	[254]
	*U. urealyticum* (58.3%)	↓Volume, sperm motility, viability	

Each publication contains information about number of bacteriospermic specimens, their origin considering fertility or infertility, with or without symptoms, identified bacteria on the species level (if specified in publication), frequency of bacterial occurrence, effect of all identified bacteria or specified to single species, and references.

## 5 Methods for the detection of bacteria in semen

Precise identification of bacterial pathogens is an essential assignment of each microbiological laboratory to aim for appropriate therapy. Conventional identification based on the Gram staining, bacterial cultures, and biochemical properties of bacterial isolates provides, in terms of accuracy, reliable results with good affordability, thanks to which these represent the “gold standard” [255]. However, their use is considerably limited by a possible nonspecific biochemical activity of microorganisms or closely related bacterial species. The usual duration of such tests requires 48–72 h for the cultivation of normally growing bacteria, while the identification of slowly growing bacteria may last for weeks [256], and nonculturable bacterial pathogens stay undetected.

Modern methods of bacterial identification retain a high sensitivity and specificity, while a small amount of sample is consumed to identify the exact species, which is particularly welcome in the diagnosis of bacteriospermia when the sample volume is often limited [176,257]. PCR-based diagnostic methods are referred to as the “new gold standard” in the molecular identification of microorganisms. At the same time, these are also widely used for the detection of virulence factors as well as resistance genes [258]. Routine PCR diagnostics has allowed to develop various modifications. Currently, quantitative PCR (qPCR) represents the most used modification in routine PCR diagnostics, which is specified by the possibility of amplifying more than one sequence during a single PCR reaction [259,260]. Real-time qPCR comes along with faster results without the necessity for additional analysis.

The success of assisted reproductive technology is particularly sensitive to the presence of bacteria. Contaminated cultures of gametes may cause damage or even loss of embryos. Therefore, a rapid and accurate bacterial screening through real-time qPCR may prevent unsuccessful fertilization [261,262]. The high sensitivity of PCR methods also allows the detection of even a low bacterial load in specimens collected from asymptomatic individuals [263].

A new milestone in microbial screening provides the next-generation sequencing. The 16 S ribosomal RNA, a component of the 30 S subunit of the bacterial ribosome, contains highly conserved and hypervariable regions (V1-V9), which allow very accurate identification and specific taxonomic classification. One limitation lies in the actual databases of the 16 S rRNA sequences [264]. Another limitation represents the financial burden of this method. Besides material and equipment, sequencing demands software for the visualization of the results, a database for comparing the obtained results, bioinformatic knowledge, and experience to design the reaction and interpret the collected data [265].

Matrix-assisted laser desorption/ionization–time-of-flight mass spectrometry (MALDI-TOF MS) has been developed for a routine application in rapid microbial diagnostics. This technique utilizes a soft ionization and separation of charged particles according to their molecular properties in magnetic and electric fields. The samples are being ionized by a laser, which is necessary to avoid thermal disintegration. For this purpose, the matrix is added to the examined sample to absorb a strong laser nitrogen beam. The molecules are transferred during excitation from the matrix to the analyte, which is then protonated or deprotonated, creating molecules with a uniform charge. Particles will pass through the vacuumed TOF tube according to their charge and mass, and the detector at the end of the tube measures the time of flight of each ion, as light ions pass faster than the heavier ones [266].

MALDI-TOF MS must contain three basic components: a source of ions and ionization to convert molecules to a gaseous state; a spectrophotometer to separate the ions based on their mass/charge ratio; and software and database to process and compare the obtained results with a database [266]. Currently, molecules ranging from 100 to 100,000 Da can be analyzed. Highly specific results are obtained by the comparison of mass peaks, which is specific for each organism. Therefore, such a protein profile has its own “fingerprint.” The only expensive investment lies in the initial costs of purchasing the machine.

According to Singhal et al. [267], only a few colonies are needed to identify microorganisms, which shortens the diagnosis by 2–3 days. A special advantage directly identifies bacteria from the collected biological material, including semen [268] and urine [269]. Moreover, an expanding area of MALDI-TOF analyses represents a rapid detection of antibiotic resistance. Currently, β-lactamase activity is measurable by MALDI-TOF MS [270].

Several studies concern the MALDI-TOF MS bacterial identification of ejaculates originating from various species, including livestock animals and human samples [30,40,53,271,272]. A rapid identification demands a purification of the sample in advance. Nevertheless, isolation and culture of pure isolates on agar plates remain the most reliable way of bacterial identification by MALDI-TOF MS [267]. It is necessary to continuously evaluate the accuracy of MALDI-TOF to improve, revise, and add new spectra to the MALDI database. A previous report compared the reliability of identification of G^+^ isolates, stating that over 92% of identifications at the species level were consistent with 16 s rRNA sequencing. Conversely, less than 86% agreed with the case of the phenotypic method [273]. Even when very rare microorganisms were identified by both methods, MALDI-TOF and 16 s rRNA sequencing provided highly accurate results at the species level [274]. Similar to the previous reports, MALDI-TOF identification is considered an accurate method providing reproducible results for identifying nonfermenting bacteria [275].

## 6 Management of bacteriospermia: limitations and challenges

Reproductive biotechnologies and assisted reproductive technologies allow preservation of the genetic material of male individuals and exploit a maximum of its fertilization potential. These techniques come along with several advantages, including genetic improvement, implementation of reproductive procedures anytime and anywhere on the planet, and the prevention of disease transmission [276]. Conversely, just the process of sperm collection and cryopreservation may be a reason for an infectious disease spread in the recipient [268,277]. Therefore, antibacterial supplements must be added to semen extenders before the freezing procedures by law regardless of any effect on the postthaw sperm quality. Even if these are used in small amounts, unspecific consumption of antibiotics leads to antibiotic resistance [278]. Since one of the main sources of bacterial contamination is the preputium, strict hygiene standards may significantly help decrease the bacterial load in neat ejaculates. The semen collection equipment itself is another important source of potential bacterial contamination. Requiring high hygiene standards during collection, processing, and storage is the most effective way to protect spermatozoa from bacterial contaminants.

Trading animal semen has demanded regulations of antimicrobial supplements in semen extenders to avoid any potential transmission of infectious diseases. Therefore, the European Union and the European Council issued regulations that clarify the use of antibiotic cocktails in each insemination dose. According to the Council directive from June 26, 1990, no less than 500 IU/mL penicillin, 500 IU/mL streptomycin, 300 µg/mL spectinomycin, and 150 µg/mL lincomycin at a final concentration must be added to avoid the spread of mycoplasmas and leptospires. According to Spinosa et al. [279], antibacterial prevention frequently relies on substances that interrupt the synthesis of the cell wall leading to cell lysis and death (β-lactams) or which inhibit bacterial proteosynthesis (aminoglycosides, lincosamides, and macrolides). As reported by several studies [280,281,282], 500 µg/mL gentamycin, 300 µg/mL lincomycin, 100 µg/mL tylosin, and 600 µg/mL spectinomycin comprise the most recommended antimicrobial cocktail. However, these authors evaluated the subsequent bacterial susceptibility 10+ years ago.

A recent report [283] has stated that over 56% of all identified bacterial species in boar semen exhibited resistance to gentamycin, 24% were intermediate, and approximately every fifth bacterium was susceptible to gentamycin, lincomycin, penicillin, and neomycin. Faisal and Salman [284] observed the prevalence of *E. coli*, *K. pneumoniae*, and *S. epidermidis* in the semen of men seeking infertility treatment, where a multidrug resistance was determined. The bacteria showed the highest resistance to gentamycin, cefotaxime, ampicillin, and levofloxacin. Contrarily, the isolated bacteria were particularly susceptible to amikacin. Gentamycin was previously considered an ineffective antibiotic supplement in several studies [38,285,286]. Despite the resistance of bacteria to penicillin, streptomycin, and sulfanilamide, which has been observed decades ago [287], these antibiotics are still being used according to valid legislation. A recently published paper on microorganisms isolated from bull semen showed an alarming pattern of antibiotic resistance: 100% of isolates were resistant to penicillin, while most isolates were resistant to tylosin and lincomycin. Moreover, the legislatively required concentrations of antibiotics were insufficient in 60% of isolated microorganisms, while only in the case of 3.9% of isolates, these concentrations were defined as satisfactory [288]. Dalmutt et al. [289] characterized bacterial contaminants of boar semen and evaluated their antimicrobial susceptibility profiles. *P. aeruginosa* and *P. mirabilis* showed the highest antibiotic resistance rate. All *P. mirabilis* isolates were resistant to spectinomycin, lincomycin, florfenicol, and streptomycin. As such, insufficient legislative regulations together with irrational antibiotic overuse may lead to an even greater deterioration in antibiotic susceptibility and an increased multidrug resistance in a wider range of microorganisms.

Density gradient centrifugation represents a method based on cell sorting according to their density. The density of an intact mature sperm cell is above 1.10 g/mL, while damaged or immature spermatozoa have a density of 1.06–1.09 g/mL. Various concentrations of a colloid are layered on the top of each interphase, while the densest colloid solution is placed on the bottom of a conical test tube, more sparse colloids are placed onto itself, and the lowest colloid concentration is found on the top of the colloid column [290]. After layering the semen sample, low-speed centrifugation separates the seminal plasma, bacteria, leukocytes, immature cells, damaged spermatozoa, residues, and intact sperm cells according to their density and accumulates them at the interface of the individual interphases.

**Figure 4:**
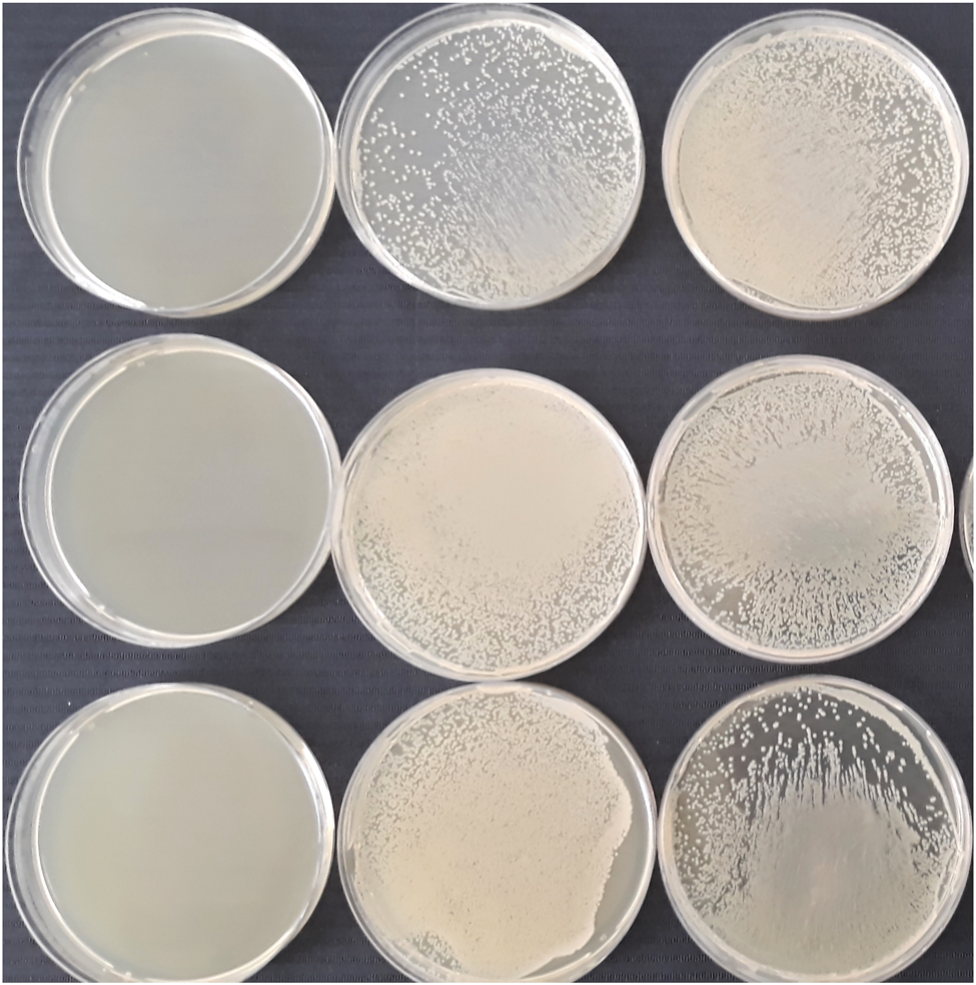
Efficacy of the density gradient centrifugation in the elimination of *S. aureus* from semen. Semen samples infected with *S. aureus* were seeded onto Tryptic soy agar. Samples placed on the left side were processed through density gradient centrifugation. No colony was grown on these plates, while the rest of the plates were overgrown by bacteria.

Previous studies have demonstrated the effective removal of bacteria and viruses from semen by several modifications of the density gradient centrifugation [278,291,292,293]. Particularly, *in vitro* fertilization practice has shown that density gradient centrifugation is an effective technique to diminish bacterial contamination [294]. Even our research team recently verified the effectiveness of bacterial removal from bovine semen (Figure 4). Besides improving the microbial status of semen, density gradient centrifugation also improves the quality of neat or thawed ejaculates of suboptimal quality. At the same time, no significant negative effects were recorded utilizing this method on normal spermatozoa. Therefore, colloid-based methods could reduce bacterial contamination without the necessity for antibiotics [295].

A recent report [39] has suggested that plant-based bioactive compounds may improve the fertility potential in semen samples contaminated with *E. faecalis*. Moreover, semen samples treated with penicillin, gentamycin, and kanamycin showed several deteriorated quality parameters compared to the experimental groups supplemented with quercetin, curcumin, and resveratrol. In particular, significant differences were observed when evaluating the sperm DNA fragmentation index. However, the potential beneficial effects of natural biomolecules on the maintenance of sperm DNA integrity were probably the result of their antioxidant properties rather than their antimicrobial activity.

Studies focused on the effects of pure bioactive substances on the microbial milieu provide promising results. However, more interesting data are oftentimes observed when evaluating the impact of plant extracts, thanks to their complex composition. Products of medicinal plants are widely used for their antioxidant effects or antibacterial properties. *Schisandra chinensis* extract ranging from 5 to 50 µg/mL exhibited outstanding protection to the sperm structures [296]. However, the minimal inhibition concentration required to inhibit the growth of 50% of the selected bacterial strains started at 64.2 µg/mL. Although the use of *Schisandra* extract maintains sperm quality on excellent levels, its use as an antimicrobial agent remains uncertain.

Elmi et al. [297] reported that *Rosmarinus officinalis* essential oil at 0.4 m/mL has exhibited antimicrobial activity comparable to ampicillin when evaluating the growth of *E. coli*, while spermatozoa quality stayed unchanged. Ros-Santaella and Pintus [298] recently reviewed plant extracts as alternative supplements for sperm preservation. Plant extracts are a relatively cheap source of beneficial substances. For example, the ginger extract at a concentration of 0.1 mg/mL has reduced the bacterial abundance in cryopreserved spermatophores, while no changes were observed in their structure or function [299]. Although several studies have published promising results on the antibacterial activity of natural bioactive compounds toward bacterial species isolated from semen, no report has revealed any pure compound or plant-based extract able to successfully diminish bacteria in semen while having beneficial effects on the sperm structure and behavior. High concentrations of bioactive substances needed to mitigate bacteriospermia on the one hand are too high to avoid a potentially negative impact on the seminal oxidative balance. In other words, effective antimicrobial concentrations of bioactive substances are too high not to act as prooxidants [300].

The use of nanoparticles may bring a new alternative to currently used antimicrobials. Boar ejaculates were previously investigated following treatment with Fe_3_O_4_ nanoparticles. After 30 min, the nanoparticles were removed, and the ejaculates were incubated for 48 h. It was revealed that the nanoparticles did not affect sperm motility, morphological characteristics, viability, membrane, and DNA integrity, while they provided a slight antimicrobial effect [301]. Silver–carbon nanoparticles were tested against bacteriospermia of fresh bovine semen. Several bacterial species, including *E. coli, S. aureus,* and *P. aeruginosa*, were isolated, and minimum inhibitory and bactericidal concentrations were analyzed. A 3.125 µg/mL concentration exhibited bactericidal activity in *S. aureus* and *P. aeruginosa*. The growth of *E. coli* was inhibited by a 12.5 µg/mL concentration. The authors have revealed that concentrations ≤30 µg/mL did not affect the sperm parameters, including motility, viability, acrosomal status, or morphology. Therefore, nanoparticles may represent a favorable option in the search for potential antimicrobial substances for stored semen [302].

## 7 Conclusions

Evidence collected in this review strongly indicates that the presence of bacteria in semen may negatively impact the sperm structure and function, leading to subfertility or even infertility. Nevertheless, the molecular mechanisms by which bacteriospermia affects male reproduction are complex and intricate. Besides a direct bacterial action on the male gamete, inflammation and oxidative stress may play pivotal roles in the pathology of bacteriospermia. Nevertheless, specific interactions of the reproductive tract and immune system during bacterial infection need further elucidation. Follow-up studies on the intricate network of relationships on a biochemical, molecular, immunological, and oxidative level may provide new directions to the development of novel diagnostic tools and biomarkers for a fast and reliable diagnosis of bacteriospermia as well as advances in appropriate strategies to prevent or manage bacterial contamination of semen in the future.
